# Case Report: Clinical characteristics and outcomes in natural killer (NK)/T-cell lymphomas with cardiac involvement: a case series and literature review

**DOI:** 10.3389/fcvm.2026.1794186

**Published:** 2026-08-20

**Authors:** Guy Robinson, Kiyan Heybati, Kevin Tayon, Rupashi Mukhia, Joseph B. Parker, William Yang, Jennifer Jiang, Jordan C. Ray, Vivienne Yang, Han Tun

**Affiliations:** 1 Department of Internal Medicine, Mayo Clinic, Jacksonville, FL, United States; 2 Department of Internal Medicine, Mayo Clinic, Rochester, MN, United States; 3 Department of Cardiovascular Medicine, Mayo Clinic, Jacksonville, FL, United States; 4 Department of Anesthesiology, Mayo Clinic, Jacksonville, FL, United States; 5 The Robert H. Lurie Comprehensive Cancer Center, Northwestern University Feinberg School of Medicine, Chicago, IL, United States; 6 Department of Laboratory Medicine and Pathology, Mayo Clinic, Jacksonville, FL, United States; 7 Department of Radiation Oncology, Mayo Clinic, Jacksonville, FL, United States; 8 Department of Haematology and Oncology, Mayo Clinic, Jacksonville, FL, United States

**Keywords:** cardiac imaging, cardiac tumour, CD30, ENKTL, extranodal natural killer/T-cell lymphoma, NKT lymphoma subtype, SMILE chemotherapy

## Abstract

**Background:**

Cardiac involvement by extranodal natural killer/T-cell lymphoma (ENKTL) and aggressive NK-cell leukaemia (ANKL) is exceedingly rare and presents with heterogeneous manifestations, often leading to diagnostic uncertainty and delay. We contribute three additional biopsy- or autopsy-proven cases and place them in the context of a literature review of previously reported cases classified according to the 5th edition of the World Health Organization (WHO) Classification of Hematolymphoid Tumors.

**Case series:**

Three men (ages 58–72) presented with diverse cardiac manifestations. A 72-year-old with progressive dyspnoea and cardiogenic shock had cMRI findings interpreted as myocarditis on an infiltrative substrate; endomyocardial biopsy confirmed cardiac infiltration by ENKTL. Despite brentuximab vedotin (BV)-based therapy he died shortly thereafter. A 60-year-old with encephalopathy, biventricular dysfunction, and widespread nodal/multiorgan disease had nodal pathology consistent with EBV-negative ANKL according to the criteria of the 5th edition of the WHO Classification of Hematolymphoid Tumors; he declined chemotherapy and died within six days, with autopsy confirming multiorgan (including myocardial and bone marrow) infiltration. A 58-year-old with prior sinonasal ENKTL treated with four cycles of SMILE developed a new right atrial mass one month later; the mass was diagnosed by cMRI/PET-CT concordance and an extracardiac (EBUS-lung) tissue confirmation of the same recurrence. BV followed by gemcitabine/oxaliplatin failed to control progression and he died approximately one month after cardiac spread. Across cases, CD56 was positive in all, EBER in two, and CD30 was expressed variably.

**Conclusions:**

Cardiac ENKTL/ANKL is a rapidly fatal entity with protean features. Multimodal imaging aids early recognition and tissue targeting, but definitive diagnosis rests on tissue confirmation. L-asparaginase-based chemotherapy remains the treatment backbone; CD30- and PD-L1-guided agents such as BV, PD-1 blockade, and daratumumab may offer transient control after biomarker-guided selection but lack randomized data in cardiac disease. Across the reported experience, survival after cardiac involvement was uniformly short, typically days to a few months. Our three cases add to this literature by illustrating the diversity of cardiac presentations, the variable CD30 expression relevant to brentuximab vedotin use, and the contribution of multimodal imaging to diagnosis. Findings from our series and the pooled literature are descriptive rather than constituting a formal survival analysis, and underscore the need for earlier diagnosis and prospective investigation.

## Introduction

Natural killer (NK)/T-cell lymphomas are rare and aggressive subtypes of non-Hodgkin lymphoma strongly associated with Epstein–Barr virus (EBV). These malignancies most commonly affect middle-aged men and predominate in East Asia and Latin America, although an increasing number of cases are being reported in Western populations (1, 2). NK/T-cell lymphomas typically arise as extranodal disease and are categorized into nasal (80% of cases, involving the upper aerodigestive tract), non-nasal (skin, gastrointestinal tract, testes, and other organs, ∼20%), and, rarely, disseminated leukaemia/lymphoma involving marrow and multiple organs (3).

Cardiac involvement is exceedingly uncommon, with only a few cases reported in the English literature (4–24). Reported manifestations include intracardiac masses (10, 13, 15, 21), diffuse myocardial thickening (5, 8), and nodular myocardial infiltration (18), leading to life-threatening complications such as arrhythmias (10, 13, 16), heart failure (14, 20, 22), or pericardial effusions (5, 15, 16). Diagnosis is challenging, as presentations can mimic myocarditis or infiltrative cardiomyopathies such as amyloidosis. Advanced imaging with cardiac MRI and PET-CT (25, 26), together with cardiac biopsy where feasible, remains essential for definitive diagnosis.

This report describes three additional cases of NK/T-cell malignancy with confirmed cardiac involvement, representing one of the few single-institution series reported to date, and contextualizes them within a literature review of previously published cases.

### Diagnostic classification and terminology

All three cases were classified according to the 5th edition of the World Health Organization Classification of Hematolymphoid Tumours (3, 27). The category of “ENKTL, nasal type” in prior classifications is retained as extranodal NK/T-cell lymphoma (ENKTL), while EBV-negative disease presenting with peripheral-blood, bone-marrow, and multi-organ involvement, a rapidly progressive course, and a mature NK-cell immunophenotype is separately designated aggressive NK-cell leukaemia (ANKL); recent genomic work supports a distinct biology, including a higher frequency of FAT-family gene mutations in ANKL relative to ENKTL with bone-marrow involvement (28). On these criteria, Cases 1 and 3 are classified as ENKTL (Case 1 with cutaneous and cardiac spread; Case 3 with sinonasal primary and cardiac relapse), and Case 2 is classified as EBV-negative ANKL based on the biventricular systolic dysfunction, multiorgan and marrow involvement (confirmed at autopsy), a mature NK immunophenotype (CD2+, surface CD3-, CD16-, CD56+, KIR CD158b+, NKG2A+, CD57-), and EBER negativity.

### Methods - literature review

We searched PubMed and Embase from January 2000 through July 2026 for English-language case reports and case series of NK/T-cell lymphoma or aggressive NK-cell leukaemia with cardiac involvement, using the strategy (“natural killer/T-cell lymphoma” OR “NK/T-cell lymphoma” OR “ENKTL” OR “aggressive NK-cell leukemia”) AND (cardiac OR myocardial OR heart OR pericardial OR endocardial). Records were screened for biopsy- or autopsy-confirmed NK-lineage lymphoma with documented cardiac involvement. Reviews without primary case data and reports of non-NK/T lymphomas were excluded. Reference lists of retrieved articles were also reviewed. Twenty-one prior cases meeting inclusion criteria are summarized in Table 1, with our three new cases added to give a pooled descriptive cohort of 24. Given the near-exclusive reliance on single case reports, statements regarding survival across the pooled cohort are presented as descriptive observations rather than formal survival analyses.

**Table 1:** Pooled cohort: previously reported cardiac ENKTL/ANKL cases plus the current series.

Ref no.	First author (Year)	Age / Sex	Primary / origin site	Cardiac manifestation	Imaging	EBV	CD30	Treatment	Outcome (survival from cardiac dx)	Notes
(4)	Abe (2012)	NR	Sinonasal	Ocular masquerade+cardiac	MRI/PET-CT	Positive	NR	NR	Died	
(5)	Baek (2014)	NR / M	Pancreatic	RV wall thickening+VT during early chemo	cMRI+PET-CT	Positive	NR	L-asparaginase-based	Died	CD56-negative ENKTL
(6)	Bayoudh (2022)	NR	Disseminated	Massive myocardial infiltration; atrial flutter+pericardial effusion	PET-CT	Positive	NR	Not specified	Died	
(7)	Farfan-Leal (2019)	NR	Cardiac (primary)	Complete AV block; right atrial involvement	cMRI+PET-CT	Positive	NR	Not specified	NR	Primary cardiac ENKTL
(8)	Goutas (2021)	NR	Cutaneous	Electromechanical dissociation; myocardial infiltration	Autopsy	Positive	Positive	Not administered / autopsy dx	Died	Autopsy series
(9)	Huang SH (2011)	NR	NK/T-cell lymphoma	Electrical storm after ICD; VT	cMRI+EMB	Positive	NR	L-asparaginase-based	Died	EMB with mononuclear infiltrate and myocyte necrosis
(10)	Kanesvaran (2009)	NR	Nasal	Malignant arrhythmia; cardiac involvement	PET-CT	Positive	NR	Not specified	Died	
(11)	Kawasaki (2008)	NR	Cutaneous	Atrial fibrillation/flutter, then cardiac arrest; conduction system infiltration	Autopsy	Positive	NR	Not specified	Died	Primary cutaneous ENKTL invading heart
(12)	Khasawneh (2024)	NR	Disseminated	Pericardial effusion with concurrent HLH	Pericardial cytology	Positive	Positive	Supportive	Died	Pericardial fluid diagnosis
(13)	Kolesnik (2022)	NR	Nasal	Cardiac relapse; incessant VT	cMRI+EMB	Positive	NR	Multimodality salvage	Alive at report	EMB-confirmed cardiac relapse
(14)	Lopez (2020)	Young M	Nasal	Acute heart failure; LV hypertrophy+diffuse LGE+pericardial thickening	cMRI+PET-CT	Positive	NR	L-asparaginase-based+supportive	Alive at report	Initial EMB non-diagnostic; extrathoracic tissue confirmed
(15)	Lepeak (2011)	NR	Cardiac (primary)	Intracardiac mass+pericardial effusion	cMRI+PET-CT	Positive	NR	Not specified	Died	Primary cardiac ENKTL
(16)	Li (2016)	NR	NK/T-cell lymphoma	Cardiorespiratory failure	cMRI+PET-CT	Positive	NR	Not specified	Died	Pericardial effusion / tamponade
(17)	Liao (2025)	NR	Nasal	Myocardial infiltration	PET-CT (diagnostic+serial)	Positive	NR	Not specified	Alive at report	PET-CT for diagnosis and therapy assessment
(18)	Marques-Piubelli (2021)	NR	Nasal	Extensive cardiopulmonary infiltration	Autopsy	Positive	Positive	Not specified	Died	Autopsy diagnosis; CD30 identified at autopsy
(19)	Thompson (2018)	NR	Nasal	Metastatic; retinal+cardiac (autopsy)	Autopsy	Positive	Positive	Not specified	Died	Ocular+cardiac autopsy findings
(20)	Xiang (2022)	NR	NK/T-cell lymphoma	Direct cardiac invasion; heart failure	cMRI+PET-CT	Positive	Negative	Not specified	Died	CD30-negative, fulminant
(21)	Yang (2020)	NR	Cardiac (primary)	Right atrial mass+pericardial nodules	cMRI+PET-CT + surgical biopsy	Positive	Negative	Chemotherapy	Died	CD30-negative
(22)	Zhang (2021)	NR	NK/T-cell lymphoma	Progressive heart failure as initial manifestation	cMRI	Positive	NR	Not specified	Died	Initially misdiagnosed as viral myocarditis
(23)	Huang D (2025)	NR	Nasal	Concurrent myocardial+CNS involvement	cMRI+PET-CT	Positive	NR	L-asparaginase-based (per report)	Died	Added in revised MS (Medicine, Baltimore)
(24)	Singh (2025)	NR	Nasal (late relapse)	Adrenal mass with suspected RA/RV + pericardial involvement	PET-CT + cMRI	Positive	NR	Salvage chemotherapy	Died	Added in revised MS (abstract in CLML); cardiac biopsy not feasible
This series - Case 1	Tun (this series)	72 / M	Cutaneous	Concentric LV hypertrophy+LGE+pericardial effusion; cardiogenic shock	cMRI+EMB	Positive	Positive (moderate)	BV + ICE; BV monotherapy	Died (within days of cardiac dx)	EMB confirmed cardiac infiltration
This series - Case 2	Tun (this series)	60 / M	Nodal / disseminated (ANKL)	Biventricular dysfunction; multiorgan Ga-67 uptake; myocardial infiltration at autopsy	Echo+Ga-67 + autopsy	Negative	Positive in subset	Declined chemotherapy	Died (∼2 days after diagnosis; ∼6 days after admission)	EBV-negative ANKL by WHO 2024; autopsy-confirmed cardiac involvement
This series - Case 3	Tun (this series)	58 / M	Sinonasal (relapsed after SMILE)	Right AV-groove mass encasing proximal RCA; RV/RA free-wall infiltration+pericardial effusion	cMRI+PET-CT + EBUS-lung biopsy	Positive	Positive (large subset)	BV then GemOx	Died ∼1 month after cardiac dx	Cardiac dx by imaging+extracardiac (lung) tissue findings concordant with same recurrence

NR, not clearly reported in the original publication. Rows marked “This series” correspond to the three index cases described in this manuscript. Two entries marked with * were identified through the systematic search described in the Methods and were not included in the original submission.

## Case 1

A 72-year-old Asian male with no known cardiovascular comorbidities presented with acute hypoxic respiratory failure following two weeks of tachycardia, tachypnoea, and a 10-pound unintentional weight loss. He had a two-month history of chronic skin ulcers unresponsive to antibiotics and episodes of epistaxis with an identified nasal polyp. On admission there was no palpable cervical or generalized lymphadenopathy, and imaging did not identify a discrete nasal mass beyond the polyp already noted (nasal-cavity involvement: polyp only; nodal involvement: absent). Initial workup focused on infection and cardiovascular causes, including concern for non-ST-segment elevation myocardial infarction and pulmonary embolism, given elevated N-terminal pro-B type natriuretic peptide (NT-proBNP) and raised but non-changing troponins. Initial laboratory evaluation demonstrated markedly elevated lactate dehydrogenase (LDH; 2,141 U/L) and ferritin (3,705 ng/mL), with a plasma EBV viral load of 6,030 copies/mL. Transthoracic echocardiography showed marked concentric left ventricular wall thickening with an apical-sparing strain pattern and a small pericardial effusion, raising suspicion for cardiac amyloidosis. Supporting features included normal-voltage ECG despite hypertrophy, proteinuria without intrinsic renal disease, and a history of bilateral carpal tunnel syndrome.

**Figure 1:**
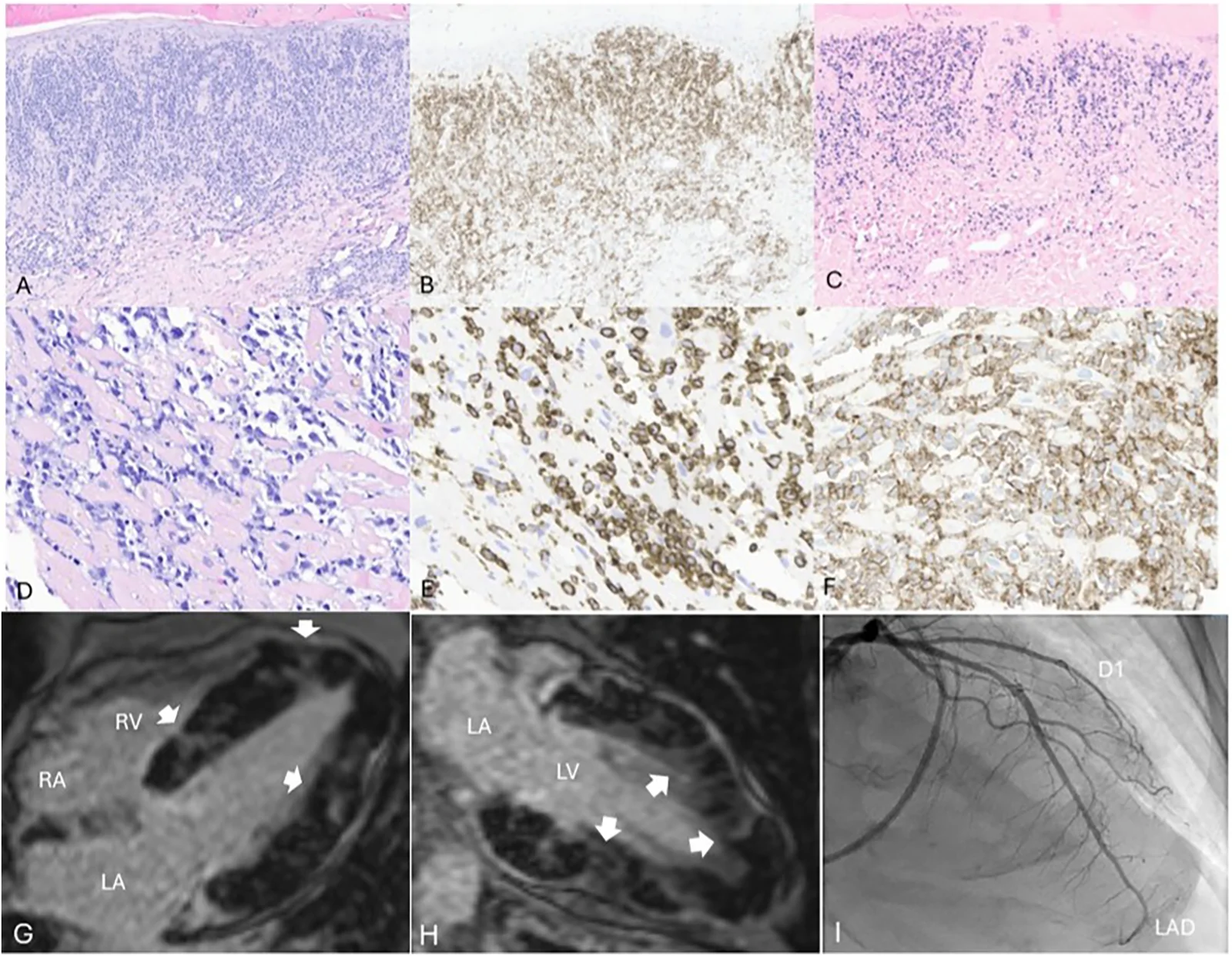
The skin biopsy showed a diffuse dermal infiltrate of neoplastic lymphocytes (**A**, H&E,  × 10). Separate single-antibody immunohistochemical stains demonstrated positivity for CD3 and CD56, with representative images shown in panel **B** ( × 10); Epstein–Barr virus-encoded small RNA *in situ* hybridization was positive (**C**,  × 10). Subsequent heart biopsy revealed an interstitial infiltrate of NK lymphoma cells (**D**, H&E,  × 40), with separate single-antibody immunohistochemical stains demonstrating positivity for CD3 (**E**,  × 40) and CD56 (**F**,  × 40). **(G,H)** Cardiac MRI demonstrates marked concentric left ventricular hypertrophy (septal thickness 21 mm, left ventricular mass index 154 g/m^2^) with diffuse subendocardial and mid-myocardial late gadolinium enhancement, increased T2 signal, and global hypokinesis sparing the apex (left ventricular ejection fraction 28%). **(I)** Coronary angiography shows no obstructive coronary artery disease, supporting a non-ischemic infiltrative process.

Laboratory evaluation revealed *λ* free light-chain elevation with a *κ*/*λ* ratio of 0.60 and a small monoclonal band on immunofixation, further raising concern for plasma cell dyscrasia and amyloid cardiomyopathy. However, skin biopsy of chronic wounds revealed a severely atypical mononuclear infiltrate involving the dermis and subcutis, with focal epidermotropism and ulceration. Immunophenotyping demonstrated CD3 + by immunohistochemistry (cytoplasmic staining pattern), CD56+, CD30+ (moderate), EBER+, and negative beta-F1 and T-cell receptor gamma/delta, consistent with ENKTL [Figure 1]. Cardiac MRI (cMRI) demonstrated marked concentric LV hypertrophy (septal thickness 21 mm, LV mass index 154 g/m^2^), depressed global function (LVEF 28%) with diffuse hypokinesis sparing the apex, and patchy regions of increased T2 signal with corresponding arterial enhancement and late gadolinium enhancement (Figure 1). These findings suggested myocardial inflammation superimposed on an infiltrative cardiomyopathy. The patient progressed to cardiogenic shock refractory to diuresis and vasopressors, requiring Impella placement for haemodynamic support.

Endomyocardial biopsy confirmed cardiac ENKTL concordant with the skin lesions [CD3 + by immunohistochemistry (cytoplasmic pattern), CD30+, CD56+, CD4-, CD5-, CD8-, CD20-]. Congo red and iron stains were negative, formally excluding amyloidosis. A staging bone marrow aspirate and biopsy showed normocellular trilineage haematopoiesis with no morphological or immunophenotypic evidence of marrow involvement (CD56-negative; flow-cytometric T-cell and KIR panels normal). Molecular/cytogenetic testing (FISH for 6q loss, 7p/17p loss, 1q gain, and NGS for FAT1) and TCR gene-rearrangement studies were not performed. Given CD30 expression, the patient was initiated on brentuximab vedotin (BV) in combination with ifosfamide, carboplatin, and etoposide (ICE). Ongoing cardiogenic shock precluded modification to a SMILE regimen (dexamethasone, methotrexate, ifosfamide, L-asparaginase, etoposide); BV was continued as monotherapy but failed to elicit a sufficient clinical response. The patient died despite maximal haemodynamic support. The endomyocardial biopsy provided direct pathology-imaging correlation with the cMRI abnormalities.

## Case 2

A 60-year-old Caucasian man with type 2 diabetes and known coronary artery disease presented with fevers, encephalopathy, profound fatigue, and a 50-pound unintentional weight loss over eight weeks. There was no nasal-cavity mass on examination or imaging. Widespread lymphadenopathy was present (mediastinal, retroperitoneal); differential diagnosis by the admitting providers included infection, autoimmune disease, and occult malignancy.

**Figure 2:**
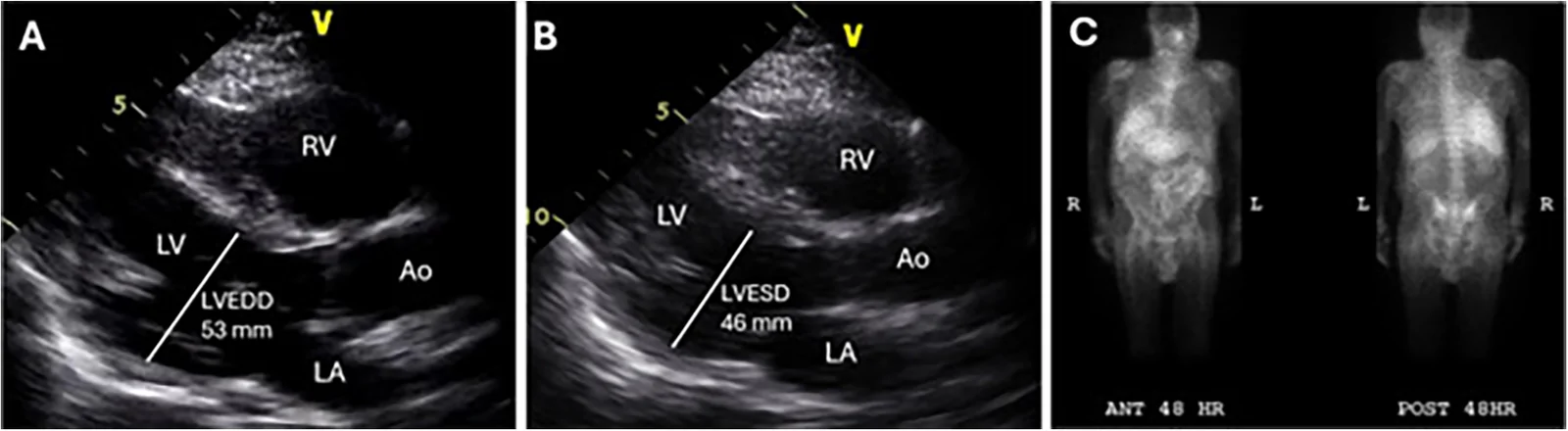
**(A,B)** transthoracic echocardiography in parasternal long-axis view during diastole **(A)** and systole **(B)** demonstrates biventricular systolic dysfunction (left ventricular ejection fraction 32%) with mild right ventricular enlargement and left ventricular dimensions of 53 mm (end-diastolic) and 46 mm (end-systolic). **(C)** Gallium-67 scintigraphy reveals multiorgan radiotracer uptake, including focal cardiac accumulation, consistent with myocardial infiltration by extranodal NK/T-cell lymphoma.

Transthoracic and transoesophageal echocardiography revealed biventricular systolic dysfunction (LVEF 32%) with mild right ventricular enlargement [Figure 2]. CT imaging demonstrated splenomegaly, mediastinal lymphadenopathy, and pulmonary embolism, prompting a brief course of anticoagulation that was discontinued due to recurrent epistaxis.

**Figure 3:**
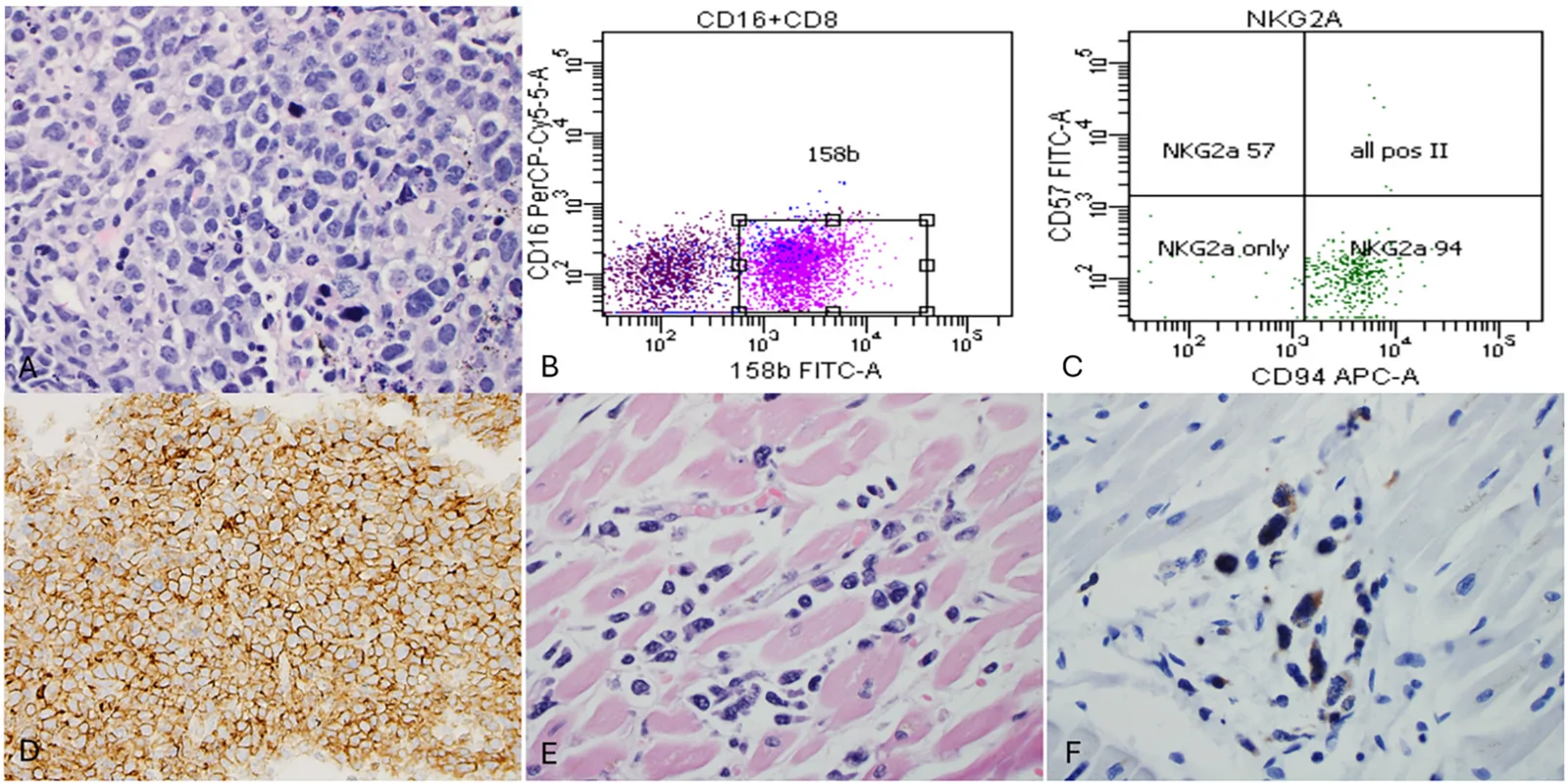
The H&E section of subcarinal lymph node fine needle aspiration showed neoplastic lymphocytes with marked nuclear atypia and frequent mitosis and apoptosis (**A**, x 40). Flow cytometry analysis identified abnormal NK cells negative for CD16 with restricted expression of CD158b, positive for CD94 and NKG2A; and negative for CD57 (the pink population in B and green in **C**). Immunostain of CD56 is strongly positive on the lymph node (**D**, x 40). The section of left ventricle from autopsy revealed small clusters of neoplastic lymphocytes in the myocardium (E, H&E x40); immunostain showed that the lymphocytes were positive for CD3 with cytoplasmic staining pattern (**F**, x 40).

Endoscopic ultrasound-guided FNA/core biopsy of a subcarinal lymph node showed sheets of medium-to-large atypical lymphoid cells with irregular hyperchromatic nuclei and frequent mitotic and apoptotic figures. Flow cytometry of the lymph-node aspirate demonstrated an abnormal NK population that was CD2+, partial CD56+, CD94+, NKG2A+, KIR CD158b+, and negative for surface CD3, CD7, CD16, CD4, CD8, and CD57. A concurrent peripheral blood flow-cytometric study identified a phenotypically similar abnormal NK-cell population, supporting circulating (leukaemic) involvement. Bone marrow biopsy was not performed. Immunohistochemistry on the nodal core confirmed CD45+, CD56+, CD43+, TIA-1+, granzyme B+, and CD30 + in a subset of cells; the diagnostic node was CD3-negative by immunohistochemistry, whereas CD3 positivity with an apparent cytoplasmic staining pattern was demonstrated by immunohistochemistry on cardiac autopsy sections. Additional stains showed strong, diffuse CD2 with negative CD25 and FoxP3, supporting an NK-cell rather than an adult T-cell leukaemia/lymphoma phenotype. EBER *in situ* hybridization was negative for EBV [Figure 3]. Based on these findings, presumptive diagnosis of EBV-negative aggressive NK-cell leukaemia (ANKL) was made. Serum LDH and ferritin values were not available in the retrospective record. Molecular/cytogenetic testing (FAT1 mutation, 6q loss, 7p/17p loss, 1q gain) was not performed; a PCR-based T-cell receptor gene-rearrangement study was attempted but was non-diagnostic owing to insufficient amplifiable DNA. Gallium-67 scintigraphy demonstrated multiorgan radiotracer uptake, including the myocardium, consistent with disseminated disease (Figure 2).

The patient declined chemotherapy and intubation. Despite ICU-level care he rapidly deteriorated and died within six days of admission. Autopsy revealed diffuse multiorgan infiltration involving lungs, liver, spleen, kidneys, pancreas, gastrointestinal tract, skin, lymph nodes, and bone marrow. Bone marrow findings confirmed the diagnosis of ANKL. Cardiac sections (left ventricle and septum) showed piecemeal myocardial infiltration by neoplastic lymphocytes with cytoplasmic CD3 positivity by immunohistochemistry, confirming myocardial involvement post mortem.

## Case 3

A 58-year-old Caucasian man with type 2 diabetes mellitus and coronary artery disease presented with progressive sinus congestion, frontal headaches, fever, night sweats, and weight loss. Facial CT imaging demonstrated extensive left sinonasal disease (nasal-cavity mass: present). Biopsy of a friable nasal mass revealed ENKTL, nasal type, EBV-positive. The excisional biopsy showed a diffuse proliferation of atypical lymphoid cells with extensive necrosis; the cells had intermediate-to-large, markedly irregular nuclei with condensed-to-vesicular chromatin and moderate cytoplasm, and mitotic figures and apoptotic bodies were readily identified. Immunophenotyping demonstrated CD3 + by immunohistochemistry (cytoplasmic staining pattern), CD30+ (large subset), CD56+ (partial and weak), TIA-1+, EBER+, and negative beta-F1, with a Ki-67 proliferation index of approximately 70%. Laboratory evaluation showed an LDH level of 244 U/L and a plasma EBV viral load of 5,580 IU/mL; a ferritin level was not available. Staging did not identify systemic nodal disease at initial presentation. Molecular/cytogenetic testing (Fish for 6q loss, 7p/17p loss, 1q gain, and NGS for FAT1) and TCR gene-rearrangement studies were not performed.

He was initiated on the SMILE regimen; during the first cycle he experienced an ST-segment elevation myocardial infarction from re-occlusion of the right coronary artery, requiring PCI. He completed four cycles of SMILE; within one month of the fourth cycle he was diagnosed with cardiac involvement.

**Figure 4:**
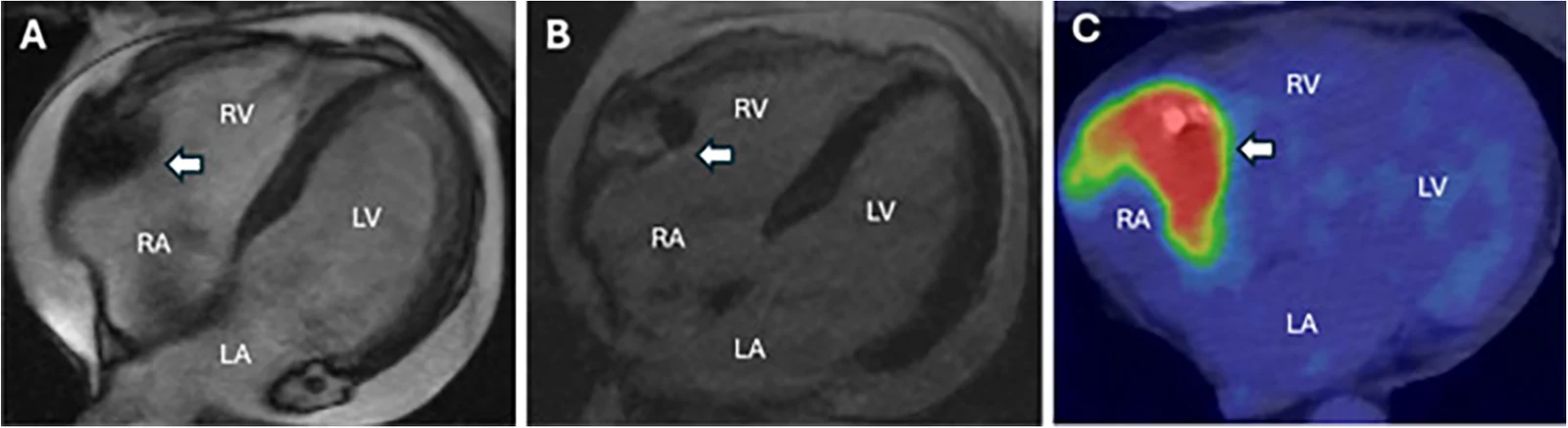
**(A,B)** Cardiac MRI reveals an irregular 5  ×  5  ×  2 cm mass centered in the right atrioventricular groove (arrow), extending into the posterior and medial right atrial wall and along the right ventricular free wall. The lesion is T2 hyperintense with mixed late gadolinium enhancement and associated moderate pericardial effusion with fibrinous strands. **(C)** Fluorodeoxyglucose positron emission tomography–computed tomography demonstrates intense radiotracer uptake within the right atrium (arrow) corresponding to the MRI-defined mass, confirming metabolically active myocardial infiltration by extranodal NK/T-cell lymphoma.

PET-CT demonstrated new FDG-avid foci in the left lung and intense uptake in the right atrium with a maximum standardized uptake value (SUV_max 15.3). Echocardiography showed abnormal increased echogenicity of the basal RV free wall and tricuspid annulus corresponding to the PET findings, with preserved LV ejection fraction (58%) but impaired longitudinal strain (−15%). Cardiac MRI defined an irregular 5  ×  5  ×  2 cm mass in the right atrioventricular groove extending into the posterior and medial right atrial wall, encasing the proximal RCA, and extending along the RV free wall [Figure 4]. The lesion was T2 hyperintense, showed mixed late gadolinium enhancement, and was associated with a moderate pericardial effusion with fibrinous deposits. LV systolic function was mildly reduced (EF 50%) with evidence of inferior wall subendocardial infarction; RV systolic function was impaired (EF 46%).

Endomyocardial biopsy was not performed because of the anatomic location of the mass and the patient's clinical trajectory. Lung biopsy via FNA/EBUS showed an atypical lymphoid infiltrate suspicious for involvement by the patient's known NK/T-cell lymphoma, with atypical cells positive for CD3 (cytoplasmic pattern by immunohistochemistry), CD30, TIA-1, and EBV, but negative for CD5, CD7, and CD56, an immunophenotype concordant with the sinonasal primary; flow cytometry on the lung specimen showed no immunophenotypic evidence of lymphoma. Bone marrow studies did not demonstrate lymphoma. Cardiac involvement in this case was therefore established by imaging concordance (PET-CT and cMRI) together with extracardiac (EBUS-lung) tissue findings concordant with the recurrent lymphoma, rather than by direct cardiac tissue confirmation.

Given CD30 expression, he was treated with BV without clinical improvement and was subsequently transitioned to gemcitabine/oxaliplatin (GemOx). Despite therapy he developed pneumonia and worsening heart failure. He died of progressive respiratory failure approximately eight months after initial diagnosis and one month after radiographic evidence of cardiac spread.

## Discussion

### Presentation

Cardiac involvement in ENKTL/ANKL demonstrates marked heterogeneity and poses a formidable diagnostic challenge. Across reported cases to date, including the three reported here, clinical presentations have ranged from incidental cardiac findings to fulminant cardiogenic shock, malignant arrhythmias, and cardiac tamponade. Despite this variability, most patients exhibited “B symptoms” such as fever, night sweats, fatigue, and unintentional weight loss, often prompting initial evaluation for infection, autoimmune disease, or occult malignancy (4, 14, 20, 21). The disease has also been reported to masquerade as pneumonia, myocarditis, or vasculitic syndromes, resulting in diagnostic delays of weeks to months (4, 9, 18).

### Electrophysiological and arrhythmic features

Electrophysiological abnormalities and malignant arrhythmias are seen in cardiac ENKTL, typically reflecting underlying myocardial infiltration or disruption of the conduction system. Ventricular arrhythmias were most common, with sustained monomorphic ventricular tachycardia (VT) reported in at least four patients (5, 9, 13, 21) and a VT storm in two (9, 20). The anatomical basis of these arrhythmias was frequently corroborated by imaging or histology. Baek et al. (5) reported RV wall thickening with heterogeneous gadolinium enhancement on cMRI and hypermetabolic activity on PET/CT coinciding with the onset of VT; in the case presented by Huang (9), endomyocardial biopsy revealed mononuclear infiltration and myocyte necrosis, confirming lymphomatous myocarditis. Atrioventricular conduction abnormalities were less common but equally ominous. Farfan-Leal et al. (7) reported complete AV block as the initial manifestation, with PET/CT and cMRI revealing right atrial involvement. Kawasaki et al. (11) described conduction system infiltration confirmed at autopsy, with the patient developing atrial fibrillation and flutter and having a cardiac arrest six days after arrhythmia appeared. In Goutas' report (8), electromechanical dissociation was the terminal event, with autopsy confirming dense myocardial infiltration and necrosis. Atrial arrhythmias were reported less frequently but remain noteworthy; Bayoudh et al. (6) described new-onset atrial flutter preceding hypotension and rapid decompensation in the context of PET-confirmed myocardial infiltration and moderate pericardial effusion. Overall, arrhythmias were often the first clinical clue of cardiac infiltration and in many cases predated confirmatory imaging or biopsy by days or weeks.

### Imaging

Cardiac imaging findings in ENKTL are heterogeneous and often non-specific. Across the three cases described, transthoracic echocardiography revealed a spectrum from concentric wall thickening with biventricular dysfunction to intracardiac mass and pericardial effusion. Pericardial effusion is common in the literature (approximately one-third of the compiled cohort), with severity ranging from small or incidental (13) to frank tamponade requiring drainage (14, 16).

Cardiac MRI can suggest myocardial infiltration but is not itself definitive. In Case 1, diffuse subendocardial enhancement and wall thickening were initially interpreted as myocarditis on a background of cardiomyopathy; endomyocardial biopsy correlated the cMRI abnormalities with direct tissue evidence of myocardial ENKTL infiltration, providing a clear imaging-pathology correlation. Similar misclassification has been reported: Zhang (22) described patchy late gadolinium enhancement originally attributed to viral myocarditis, and Lopez (14) attributed LV hypertrophy, diffuse enhancement, and pericardial thickening to Coxsackie myocarditis before extracardiac tissue established ENKTL.

Importantly, direct cardiac tissue confirmation of myocardial infiltration was obtained only in Case 1 (endomyocardial biopsy). In Case 2, cardiac involvement was inferred ante mortem from multiorgan Gallium-67 uptake and confirmed only at autopsy, not by ante-mortem endomyocardial biopsy. In Case 3, cardiac involvement was established by imaging concordance (PET-CT and cMRI) with extracardiac (EBUS-lung) tissue findings concordant with the same recurrent lymphoma; no endomyocardial biopsy was performed. Statements in this manuscript regarding imaging patterns that “mimic myocarditis” or “mimic amyloidosis” therefore relate primarily to Case 1, in which direct tissue-imaging correlation was available.

By contrast (18), F-FDG PET/CT is the most sensitive modality for systemic staging and can reveal metabolically active cardiac disease even when anatomic imaging is non-diagnostic (29–33). In this series, PET/CT was decisive in Case 3 (hypermetabolic right atrial lesion, SUV_max 15.3) and has similarly exposed occult cardiac involvement in prior reports (5, 10, 17). PET/CT also supports response assessment using Deauville and volumetric metrics [SUV_max, total metabolic tumour volume [TMTV], and total lesion glycolysis [TLG]] (32–35), although prognostic implications specific to cardiac involvement remain undefined.

Tissue diagnosis is essential to confirm ENKTL/ANKL with cardiac involvement and to distinguish it from the inflammatory cardiomyopathies it frequently mimics; sampling can be endomyocardial, pericardial, or extracardiac. In this series, one patient was diagnosed by urgent endomyocardial biopsy, one only at autopsy after multiorgan Ga-67 findings, and one by EBUS-guided lung biopsy concordant with cMRI/PET findings. Similar approaches are described in the literature: Kolesnik et al. (13) confirmed cardiac relapse by endomyocardial biopsy after multimodality imaging; Yang et al. (21) achieved diagnosis by open surgical biopsy of epicardial/pericardial nodules; and Kawasaki et al. (11) reported a primary cutaneous NK/T-cell lymphoma directly invading the heart, diagnosed on skin tissue. Notably, biopsy is not infallible: Lopez et al. (14) reported a non-diagnostic endomyocardial biopsy in a patient later confirmed to have ENKTL on extrathoracic tissue. Together, these reports argue for early tissue sampling (cardiac or extracardiac) interpreted alongside PET/CT and cMRI to minimize diagnostic delay and misclassification.

### Differential diagnosis of infiltrative cardiac disease

Because cardiac ENKTL/ANKL rarely presents with a pathognomonic imaging signature, the clinician's task is to distinguish it from the more common infiltrative and inflammatory mimickers. In our Case 1, the initial differential included cardiac amyloidosis on the basis of apical-sparing strain, normal-voltage ECG despite hypertrophy, and bilateral carpal tunnel syndrome; however, Congo red staining of the cardiac tissue was negative and endomyocardial biopsy confirmed lymphomatous infiltration. In practice, cardiac amyloidosis can be non-invasively supported by grade 2–3 myocardial uptake on ^99^mTc-pyrophosphate scintigraphy for transthyretin amyloidosis (48) and requires biopsy plus mass spectrometry for typing; apical-sparing strain and small ventricular volumes with concentric hypertrophy are supportive but non-specific (49). Viral or lymphocytic myocarditis characteristically shows subepicardial or mid-wall late gadolinium enhancement, T2-signal edema, and endomyocardial-biopsy Dallas-criteria lymphocytic infiltrate; the updated Lake Louise criteria improve non-invasive sensitivity but do not replace tissue confirmation, particularly when malignant infiltration is a competing diagnosis (50). Cardiac sarcoidosis produces patchy mid-myocardial or transmural LGE and focal FDG-PET uptake against a suppressed background achieved by a high-fat/no-carb preparation, together with non-caseating granulomas on tissue (per Heart Rhythm Society consensus) (51). Primary cardiac lymphoma is most often diffuse large B-cell lymphoma involving the right atrium, in older patients or under immunosuppression; imaging typically shows a right-heart-predominant infiltrating mass, and immunophenotyping distinguishes B-lineage tumours from ENKTL/ANKL (52). Two features can help point away from these mimickers and toward NK/T-cell malignancy: (i) an aggressive, extranodally-dominant systemic pattern on PET/CT with concurrent skin, sinonasal, or gastrointestinal lesions, and (ii) an NK-lineage immunophenotype on any accessible tissue site, particularly CD56 positivity with cytoplasmic CD3, EBER, and cytotoxic granule proteins.

### Histopathologic and immunophenotypic features

NK/T-cell lymphomas are typically characterized by CD56 positivity, CD3*ε* expression by immunohistochemistry, EBER positivity, and expression of cytotoxic granule proteins such as TIA-1, perforin, and granzyme B. The classical NK-lineage phenotype is described as surface CD3-negative and cytoplasmic CD3*ε*-positive. However, routine paraffin-section CD3 immunohistochemistry cannot reliably distinguish cytoplasmic from surface expression. Direct assessment of surface CD3 requires flow cytometry or testing of fresh or frozen tissue. Table 2 summarizes the immunophenotype of our three cases, including the method by which CD3 expression was assessed, cytotoxic markers, KIR/NKG2A, and Ki-67, with markers that were not tested in the clinical work-up explicitly shown as ‘not performed'.

**Table 2:** Per-case immunophenotype and diagnostic work-up.

Marker	Case 1	Case 2	Case 3
Age/Sex	72/M	60/M	58/M
Diagnosis (WHO 2024)	ENKTL (extranodal NK/T-cell lymphoma)	Aggressive NK-cell leukaemia (ANKL), EBV-negative	ENKTL, nasal type (relapsed with cardiac involvement)
EBER (EBV)	Positive	Negative	Positive
Surface CD3	Not assessed (IHC only)	Negative (flow, absent on surface)	Not assessed (IHC only)
Cytoplasmic CD3 (IHC)	Positive (heart and skin biopsy)	Positive (cardiac autopsy); negative on nodal IHC	Positive (nasal biopsy and lung biopsy)
CD2	Not performed	Positive	Negative
CD4	Negative	Negative	Negative
CD5	Negative	Negative	Negative
CD7	Not performed	Negative (flow)	Negative
CD8	Negative	Negative	Negative
Beta-F1 (*β*F1)	Negative	Not performed	Negative
TCR *γ*/*δ* (protein)	Negative	Negative (flow)	Not performed
CD16	Not performed	Negative	Not performed
CD20	Negative	Negative	Negative
CD30	Positive (moderate)	Positive in a subset	Positive (large subset)
CD43	Negative	Positive	Negative
CD45	Not performed	Positive	Not performed
CD56	Positive	Positive (partial)	Positive (partial/weak; lost at lung recurrence)
CD57	Not performed	Negative	Not performed
CD94	Not performed	Positive	Not performed
ALK	Not performed	Not performed	Negative
NKG2A	Not performed	Positive	Not performed
KIR (CD158b)	Not performed	Positive (CD158b+, restricted)	Not performed
TIA-1	Not performed	Positive	Positive
Granzyme B	Not performed	Positive	Not performed
Perforin	Not performed	Not performed	Not performed
Ki-67 (%)	Not documented	Positive (MIB-1; not quantified)	∼70%
TCR gene rearrangement	Not performed	Attempted; non-diagnostic (insufficient DNA)	Not performed
Cytogenetics/NGS	Not performed	Not performed	Not performed

Cytoplasmic CD3 (by immunohistochemistry) and surface CD3 are reported separately; surface CD3 was directly assessed by flow cytometry only in Case 2. Markers not tested in the clinical work-up are shown as “not performed.” Molecular/cytogenetic were not performed in any of the three cases. TCR rearrangement studies were not performed in cases 1 and 3.

Our series largely reflected the expected immunophenotype, though with important variability. All three patients demonstrated CD56 positivity. Two were EBER-positive; one (Case 2) was EBV-negative and satisfied the criteria for aggressive NK-cell leukaemia according to the 5th edition of the World Health Organization Classification of Hematolymphoid Tumors. In Cases 1 and 3, the neoplastic cells were CD3-positive by immunohistochemistry, with an apparent cytoplasmic staining pattern; surface CD3 expression was not directly assessed. In Case 2, the diagnostic lymph node was surface CD3-negative by flow cytometry and CD3-negative by immunohistochemistry, whereas cardiac autopsy sections demonstrated CD3 positivity by immunohistochemistry with an apparent cytoplasmic staining pattern. Across the published cardiac ENKTL cases, cytoplasmic versus surface CD3 status was explicitly documented in only a handful of reports (10, 11, 15, 16, 21, 22), and many did not specify which form of CD3 was assessed, limiting interpretability. EBV-negative disease, such as our Case 2, can behave similarly aggressively, consistent with prior observations that CD56- or EBV-negative NK-lineage tumours may follow a comparable clinical course when cytotoxic markers are present (36).

CD30 expression, increasingly relevant for therapeutic decision-making, was inconsistently reported in the literature and most often identified at autopsy (12, 18, 19). In this series, all three patients demonstrated some degree of CD30 expression; the exact percentage of tumour cells expressing CD30 was not quantified in Cases 1 and 3 (reported as “moderate” and “large subset” respectively) and was reported as “positive in a subset” in Case 2. This inconsistency is characteristic of the literature. In Cases 1 and 3, CD30 positivity guided the use of BV-based therapy, albeit with limited efficacy; in Case 2, CD30 positivity was noted but the patient declined treatment. CD30 negativity has been explicitly described in at least two published cases (20, 21), both associated with fulminant disease. Overall, the variability of CD30 expression observed here is consistent with broader studies of ENKTL, in which CD30 positivity ranges from approximately 22% to 77% (43–45).

### Literature review

Table 1 summarizes the 21 previously reported cardiac ENKTL/ANKL cases identified through our search, together with our three index cases (pooled *n* = 24). Descriptively, the pooled cohort was predominantly male (approximately 80%) with a median age in the sixth decade; EBV was positive in the vast majority of reported cases, with EBV-negative disease exemplified by our Case 2 falling into the ANKL category. Cardiac manifestations included intracardiac masses (most often right atrial), diffuse or nodular myocardial infiltration, pericardial effusion or tamponade, and conduction-system involvement. Direct cardiac tissue confirmation (endomyocardial biopsy or surgical cardiac biopsy) was reported in a minority of cases, with the diagnosis more commonly established by extracardiac tissue plus imaging concordance or by autopsy. Outcomes were poor across the cohort: the majority of patients died within weeks to months of the cardiac diagnosis, with only a small number reported alive at the time of publication. Given that essentially all reports are single cases without standardized follow-up, any statement about “overall survival” in cardiac ENKTL should be understood as a descriptive observation drawn from case reports rather than a formal survival analysis; publication and selection bias likely favour reporting of both extreme (rapidly fatal) and unusual (long-surviving) presentations.

### Treatment

There are no established standard therapies specifically for ENKTL or ANKL with cardiac involvement, and no randomized trials have addressed this population. Recommendations below are extrapolated from trials and consensus guidance for advanced or extranodal ENKTL and ANKL in patients without explicit cardiac involvement.

Anthracycline-based regimens such as CHOP are both ineffective in ENKTL due to intrinsic multidrug resistance and contraindicated in many patients with reduced cardiac function, given the dose-dependent cardiotoxicity of anthracyclines, particularly in those with pre-existing compromise (37). Current expert consensus for advanced or extranodal ENKTL favours non-anthracycline, L-asparaginase-containing multi-agent regimens as the first-line therapeutic backbone, including SMILE (steroid, methotrexate, ifosfamide, L-asparaginase, etoposide), DDGP (dexamethasone, gemcitabine, cisplatin, pegaspargase), AspaMetDex (L-asparaginase, methotrexate, dexamethasone), P-GEMOX (pegaspargase, gemcitabine, oxaliplatin), and IMEP-L-asp; the L-asparaginase component is critical, reflecting tumour dependence on exogenous asparagine (2, 3, 38). Pegaspargase and L-asparaginase are FDA-approved for lymphoid malignancies, although not specifically for cardiac ENKTL (39). In our series, Case 3 was treated first-line with SMILE prior to cardiac relapse, and Case 1 was unable to receive intensive asparaginase-based induction because of ongoing cardiogenic shock, a common scenario in cardiac disease. When cardiac stability allows, aggressive L-asparaginase-based induction should be pursued in preference to CD30- or PD-1-directed monotherapy.

The role of hematopoietic stem cell transplantation (HSCT) is well established in advanced or high-risk ENKTL and in ANKL; autologous HSCT is commonly used to consolidate first complete remission in advanced ENKTL, and allogeneic HSCT is considered for ANKL and for relapsed/refractory disease, with cohort data reporting 4-year overall survival of ∼50% in transplanted ENKTL patients, though response at transplant is the dominant prognosticator (53, 54). None of our three patients were candidates for HSCT because of rapid clinical deterioration, cardiogenic shock, or patient preference to forgo intensive therapy.

Among biomarker-directed agents, brentuximab vedotin (BV), an anti-CD30 antibody-drug conjugate, has demonstrable activity in CD30-positive peripheral T-cell lymphomas but is not standard therapy for ENKTL: CD30 expression in ENKTL is variable (approximately 22%–77% positivity in published series), no phase III trial supports BV in cardiac ENKTL, and its role in this setting is therefore investigational and confined to salvage in biomarker-selected patients (40–42). In our series BV was administered in the two CD30-positive treated patients (Cases 1 and 3) after confirmation of CD30 expression on tissue; neither achieved a durable response. These outcomes align with prior reports that BV monotherapy in ENKTL can provide transient control but rarely durable benefit once disease is aggressive and systemic. Combinations of BV with L-asparaginase-based chemotherapy or radiotherapy may warrant prospective study, but current evidence in cardiac ENKTL is insufficient to recommend BV as anything other than a CD30-guided salvage option.

PD-1 blockade has shown activity in relapsed/refractory ENKTL: pembrolizumab monotherapy has produced overall response rates of approximately 33%–57% in single-institution studies and prospective cohorts (55, 56), and the ORIENT-4 phase 2 trial of sintilimab reported an ORR of approximately 75% with encouraging survival in relapsed/refractory disease (57). To date, however, no published data specifically examine checkpoint inhibitors in cardiac ENKTL, where rapid deterioration frequently precludes trial enrolment. In addition, ICI use in a patient with pre-existing myocardial infiltration warrants particular caution because immune-related myocarditis is uncommon but carries a fatality rate of approximately 50% and can be difficult to distinguish clinically from progression of lymphomatous myocarditis (58). Any PD-1 use in cardiac ENKTL should therefore be biomarker-guided [PD-L1 expression, which is reported in 56%–80% of ENKTL cases (46, 47)] and paired with prospective cardiac monitoring.

Anti-CD38 monoclonal antibody therapy is an emerging option. In an open-label phase 2 trial of daratumumab monotherapy in relapsed/refractory ENKTL, Huang et al. reported an objective response rate of 25% and manageable toxicity, although no complete responses were achieved and response duration was short (59). A recent case report of EBV-negative ANKL treated with daratumumab added to CHOP described sustained remission for more than 18 months, providing early signal in the ANKL subset in which our Case 2 would fall (60). These agents deserve further study in cardiac ENKTL/ANKL.

Overall, cardiac ENKTL/ANKL represents an extraordinary management challenge. Patients frequently present with cardiovascular instability that precludes immediate intensive systemic therapy. Reported longer-term survivors have in common early detection of cardiac infiltration (often via PET/CT), timely tissue confirmation, sufficient physiologic reserve to tolerate uninterrupted L-asparaginase-based chemotherapy, and multidisciplinary supportive care including antiarrhythmic control and, when necessary, mechanical circulatory support (10, 13, 14, 17). A pragmatic sequencing framework in stable patients is: (i) confirm tissue diagnosis and lineage including cytoplasmic CD3, CD56, EBER, and cytotoxic markers, and CD30 and PD-L1 expression; (ii) initiate L-asparaginase-based induction (e.g., SMILE or DDGP) with concurrent multidisciplinary cardio-oncology support; (iii) plan for HSCT consolidation in eligible responders; and (iv) reserve BV, PD-1 blockade, or daratumumab for biomarker-selected salvage or investigational combinations, acknowledging that no randomized data currently support any of these agents in cardiac ENKTL.

### Limitations

Several limitations should be acknowledged. First, this is a single-institution retrospective series of three patients, and our pooled cohort of 24 cases comprises predominantly single case reports; conclusions regarding presentation, imaging patterns, and outcome are therefore descriptive rather than statistically inferential, and are subject to publication and selection bias. Second, molecular and cytogenetic testing (FISH for 6q loss, 7p/17p loss, 1q gain; NGS for FAT1 mutations) was not performed in any of our three cases, and a T-cell receptor gene-rearrangement study was attempted only in Case 2, where it was non-diagnostic owing to insufficient amplifiable DNA; diagnostic classification according to the 5th edition of the World Health Organization Classification of Hematolymphoid Tumors was based on morphology, immunophenotype (surface versus cytoplasmic CD3, CD56, cytotoxic markers), and EBER status, together with the clinical picture. Third, direct cardiac tissue confirmation of myocardial infiltration was obtained ante mortem only in Case 1, with Case 2 confirmed at autopsy and Case 3 established by imaging concordance and extracardiac tissue; specific imaging-pathology correlations therefore have differing strength across cases and are highlighted as such. Fourth, no randomized trial data exist specifically for cardiac ENKTL, and therapeutic recommendations here are extrapolated from studies of advanced or extranodal ENKTL and ANKL without explicit cardiac involvement.

## Conclusion

Cardiac involvement in ENKTL and ANKL represents a rare but life-threatening complication associated with poor prognosis. Our series and pooled literature review highlight the heterogeneity of clinical presentations, the diagnostic challenges (especially the mimicry of amyloidosis, myocarditis, sarcoidosis, and primary cardiac lymphoma), and the limited therapeutic options currently available. Standardized reporting of surface versus cytoplasmic CD3 status, CD30 quantification, and PD-L1 expression across future case reports would materially improve pooled interpretation. Future research should focus on prospective evaluation of biomarker-directed agents (BV, PD-1 blockade, daratumumab) in combination with L-asparaginase-based induction and HSCT consolidation in patients with cardiac involvement.

## Data Availability

The original contributions presented in the study are included in the article/Supplementary Material, further inquiries can be directed to the corresponding author.
